# Reactivity of non-organometallic ruthenium(II) polypyridyl complexes and their application as catalysts for hydride transfer reactions

**DOI:** 10.3389/fchem.2023.1150164

**Published:** 2023-03-15

**Authors:** Marta Chrzanowska, Anna Katafias, Rudi van Eldik

**Affiliations:** ^1^ Faculty of Chemistry, Nicolaus Copernicus University in Toruń, Toruń, Poland; ^2^ Department of Chemistry and Pharmacy, University of Erlangen-Nuremberg, Erlangen, Germany

**Keywords:** NAD^+^ reduction, multiphase substitution, homogeneous catalysis, hydride transfer, reductive stress

## Abstract

Recently, we investigated the substitution behavior of a series of ruthenium(II) complexes of the general formula [Ru^II^(terpy)(N^∧^N)Cl]Cl, where terpy = 2,2′:6′,2″-terpyridine, N^∧^N = bidentate ligand, in aqueous solutions. We have shown that the most and least reactive complexes of the series are [Ru^II^(terpy)(en)Cl]Cl (en = ethylenediamine) and [Ru^II^(terpy)(phen)Cl]Cl (phen = 1, 10-phenantroline), respectively, as a result of different electronic effects provided by the bidentate spectator chelates. Polypyridyl amine Ru(II) complex, *viz.* [Ru(terpy)(en)Cl]Cl and [Ru(terpy)(ampy)Cl]Cl (where ampy = 2-(aminomethyl)pyridine), in which the terpy chelate labilizes the metal center, are able to catalyze the conversion of NAD^+^ to 1,4-NADH using sodium formate as a source of hydride. We showed that this complex can control the [NAD^+^]/[NADH] ratio and potentially induce reductive stress in living cells, which is accepted as an effective method to kill cancer cells. Polypyridyl Ru(II) complexes, characterized in terms of the behavior in aqueous solutions, can be used as model systems to monitor heterogeneous multiphase ligand substitution reactions at the solid-liquid interface. Colloidal coordination compounds in the submicron range were synthesized from Ru(II)-aqua derivatives of starting chlorido complexes *via* the anti-solvent procedure and stabilized by a surfactant shell layer.

## Introduction

Antitumor properties of cis-platinum discovered in the mid-60s of the last century opened up a new possibility for cancer chemotherapy. Currently, three platinum(II) coordination compounds—cisplatin, carboplatin, and oxaliplatin—are chemotherapeutic agents worldwide (Zhang et al., 2022). Side effects, the phenomenon of drug resistance, and the narrow spectrum of action accompanying the treatment of platinum drugs prompted scientists to constantly search for new transition metal complexes with anticancer properties (Oun et al., 2018). The interest in ruthenium complexes in biological research has led to the introduction into clinical trials of three simple ruthenium(III) compounds named NAMI-A, KP1019, and its more soluble analog, KP1339. These compounds have become desirable potential candidates for anticancer drugs due to the discovery of their anticancer activity in the metastatic phase and as cytotoxic agents for treating platinum-resistant colorectal cancers (Chang et al., 2016; Alessio and Messori, 2019). Compared to the platinum complexes, the mechanism of action of ruthenium compounds tested as anticancer drugs is much less known. The concept of the mechanism of action is that they are pro-drugs activated by reducing Ru(III) to Ru(II) by biologically available reducing agents—ascorbic acid or glutathione (Zhang and Sadler, 2017). Based on this knowledge, scientists focused on searching for ruthenium(II) complexes with promising biological properties. Particularly noteworthy is the area of Ru(II) complex with the formula [(η^6^-bip)Ru(en)Cl]^+^ (RM175), where bip = biphenyl, en = ethylenediamine. Obtained in 2001, RM175 was one of the first Ru(II) complexes tested for its antitumor activity (Lin et al., 2018; Coverdale et al., 2019). Organometallic ligands stabilize the oxidation state of the metal center, preventing its oxidation to Ru(III). Experimental and theoretical observations confirmed a strong preference for the selective binding of RM175 to N7 guanine, similar to platinum(II) complexes used in medicine (Noffke et al., 2012). The complex shows promising antitumor activity *in vitro* and *in vivo*, also against platinum drug-resistant tumor cells. The activity of the RM175 complex against the A2780 human ovarian carcinoma cell line is comparable to that of carboplatin (Noffke et al., 2012; Babak et al., 2015). Modifications of the coordination sphere led to the discovery of half-sandwich Ru-arene complexes. Discovered in 2004, Ru-arene-PTA compounds (RAPTA) with the general formula [Ru(arene)(PTA)X_2_], where PTA = 1,3,5-triaza-7-phosphatricyclo-[3.3.1.1]-decane and X = halogenide or biscarboxylate, showed favorable anticancer properties in preclinical studies (Nabiyeva, 2020). The presence of the PTA moiety causes better solubility in aqueous solutions and the selective activation of the complexes in tumor cells, characterized by hypoxic conditions. Under low pH, the PTA group is easily protonated to the active form, which can interact with DNA and proteins (Lee et al., 2020). Interestingly, RAPTA complexes showed a similar spectrum of activity to NAMI-A despite differences in oxidation state, ligands, charge, and geometry (Chow et al., 2016; Anuja et al., 2022).

However, the type of metal, its oxidation state, and the type of ligands determine the complexes’ biological activity and mechanism of action. Complexes with different chemical, physical and biological properties can be obtained by modifying the coordination sphere.

Modifying the coordination sphere of Ru-arene complexes led to the formation of promising potential anticancer compounds with an unconventional mechanism of action. The catalytic oxidation of glutathione (GSH) to glutathione disulfide (GSSG) introduces a new concept for the design of transition metal complexes with a different mechanism of action than cisplatin, which may allow the maintenance of low and safe therapeutic doses with minimal side effects. Designed by Sadler and co-workers, organometallic complexes of the general formula [(η^6^-arene)Ru(azpy)I]^+^, where azpy = 2-(phenyloazo)pyridine may play an important role in the redox biology of a cell by producing reactive oxygen species (ROS). Inducing oxidative stress is an effective method of killing cancer cells. An increase in ROS levels disrupts redox homeostasis within cells and causes their destruction (Soldevilla-Barreda et al., 2015).

Further modification of the coordination sphere allows for obtaining another group of compounds, the properties of which can be helpful in anticancer therapy. Introducing the N, N-donor chelate sulfonamide ligand into the coordination sphere influences the mechanism of action of organometallic Ru(II) compounds. Ruthenium(II)-arene complexes of the type [(η^6^-arene)Ru(N^∧^N)Cl]^+^, where N^∧^N = N, N-donor chelating ligands with sulfonamide substituents of various steric and electronic properties, catalyze the hydride transfer reaction to NAD^+^ in the presence of formate, glycerol, isopropanol, and hydrogen as its source (Soldevilla-Barreda et al., 2015; Chen et al., 2018). Thus, these complexes can catalytically reduce NAD^+^ to NADH, inducing reductive stress by controlling the [NAD^+^]/[NADH] ratio (Soldevilla-Barreda et al., 2015; Chen et al., 2018; Chrzanowska et al., 2020a; Inverti and Sadler, 2020).

Redox pairs NAD^+^/NADH, NADP^+^/NADPH, GSSG/GSH are necessary to maintain redox homeostasis in cells and modulate cellular metabolism. These biological redox buffers serve as cofactors or substrates for the enzymatic or non-enzymatic neutralization of reactive oxygen species to maintain a relatively reducing environment within cells. In addition, being responsible for the transfer of electrons, they play an important role in the processes of energy production in cells. The balance between the reduced and oxidized forms within each redox pair is a prerequisite for proper metabolism. Disruption of this balance leads to redox, reductive or oxidative, stress, and cell dysfunction (Xiao and Loscalzo, 2020). When, there is excessive production of reactive oxygen and nitrogen species (RONS) in cells or a decrease in the concentration of available antioxidants, one refers to oxidative stress (OS). Excessive production of antioxidants leads to reductive stress (RS). Both reductive and oxidative stress (collectively referred to as redox stress) can promote the production of RONS, leading to oxidative damage to macromolecules and disruption of cellular functions (Pérez-Torres et al., 2017; Xiao and Loscalzo, 2020). While oxidative stress has been extensively studied (Schieber and Chandel, 2014), knowledge about the underlying mechanisms of reductive stress and its biological consequences, as well as how cells respond to reductive stress, is still limited. Since organometallic ruthenium(II) compounds can affect the NAD^+^/NADH balance, they may play an important role in the redox biology of cells.

Recently, it has been shown that a similar effect can be achieved using non-organometallic complexes of the general formula [Ru(terpy)(N^∧^N)Cl]^+^, where terpy = 2,2′:6′,2″-terpyridine, N^∧^N = ethylenediamine or 2-(aminomethyl)pyridine (Chrzanowska et al., 2020a; Chrzanowska et al., 2022). Organometallic complexes used, by Sadler et al., as hydride transfer catalysts contained an aromatic ligand in the inner-coordination sphere, e.g., cyclopentadienyl (Cp) or its methyl derivative (Cp*). Since the metal-arene bond is generally very strong, a complex with such a ligand is usually highly stable. At the same time, the central ion-carbon bond and other spectator ligands significantly impact the reactivity of these compounds, i.e., the ease of substitution of the monodentate ligand present in the coordination sphere, which is important in modulating the catalytic abilities of the complexes. In the case of Ru(II) polypyridyl complexes, 2,2′:6′,2″-terpyridine plays a role similar to the aromatic ligand. Compared to organometallic complexes, the advantage of inorganic coordination compounds is their biocompatibility, known for many related systems currently used in anticancer therapy. Organometallic complexes often do not have this property. Many of them are not soluble in an aqueous solution. Moreover, they are not stable in aqueous solutions, they need to be generated *in situ*, unlike non-organometallic Ru(II) complexes, which can be prepared and isolated in advance (Betanzos-Lara et al., 2013; Chrzanowska et al., 2020a; Inverti and Sadler, 2020).

One of the rapidly developing fields of chemistry in recent decades is nanochemistry. Since nanomaterials often differ markedly from those of larger particles of the same chemical, they have received considerable attention from material scientists and have a large range of potential and actual applications. Medicinal applications of nanomaterials (nanoparticles, NP, and nanoscale coordination compounds, NSCC) cover a wide area, such as the inhibition of HIV-1 infection, nano-encapsulation engineering, antibacterial properties, antioxidant nanoparticles, nanoparticle carriers for coordination complexes, and nanoscale drug delivery (Spokoyny et al., 2009; Beloglazkina et al., 2012; Montazerozohori et al., 2014; Montazerozohori et al., 2015; Sheikhshoaie and Tohidiyana, 2015; Wu et al., 2016).

It is especially their biological application through the encapsulation of NP and NSCC by degradable polymers for drug delivery with only a partial understanding of the underlying reaction mechanisms that has received considerable attention (Imaz et al., 2009; Amorin-Ferre et al., 2013; Novio et al., 2013; Nador et al., 2014; Novio et al., 2014; Borges et al., 2015; Jans et al., 2016).

It is well known that the size and surface structures of nanomaterials are the main factors characterizing their physical and chemical properties. In recent years, the principles of coordination chemistry have been applied to understand the binding of ligands to the surface of metal nanomaterials and to describe their displacement by stronger nucleophiles in ligand substitution reactions (Boles et al., 2016; Liu et al., 2017).

However, despite the ongoing fast development of nanomaterials science, knowledge about the chemical behavior, modification, and tuning of properties of transition metal nanoparticles and nanoscale coordination compounds, is very limited. In terms of the surface coordination chemistry of nanomaterials, the emphasis is on how ligands are coordinated to the surface of the metal atoms and how they influence the properties of nanomaterials at a molecular level. The difficulty in studying the surface of nanomaterials of coordination compounds consists mainly of the lack of effective tools for characterizing their structure-surface properties. The mechanistic understanding of chemical reactions at the interface of multiphase chemical processes involving NP and NSCC has received little experimental attention and is mostly handled by computational techniques. This is because the heterogeneous nature of such reactions occurring at the liquid-solid interface cannot be treated and understood at the molecular level. However, the available results show a strong link between surface coordination chemistry and different surface properties of metal nanomaterials (Langford et al., 2018). Following, the principles of coordination chemistry, the functional properties of nanomaterials can be affected by ligand exchange reactions on the surface of such materials. Moreover, ligand exchange reactions at the liquid-solid interface may be important in the activation of nano-coordination compounds, which is important from the point of view of their potential biological application. Surface ligands coordinated to metal nanomaterials can promote their catalytic activity *via* steric and electronic interactions. In recent times, van Eldik et al. published pioneering work on heterogeneous multiphase ligand substitution reactions (Langford et al., 2018). The group provided spectroscopic evidence for such reactions at the solid-liquid interface of a sub-micrometer gold(I) carbene complex and demonstrated their efficiency. In this model solid-state system, a chloride ligand coordinated to the Au(I) center was systematically and reproducibly displaced by iodide from the surrounding solvent in which the submicron particles of the complex were insoluble (Langford et al., 2018).

In this paper, we report the results of our studies on a range of applications of Ru(II) polypyridyl complexes with various electronic properties (σ-donor and π-back bonding) and steric hindrance provided by spectator ligands as a catalyst in the regio-selective transfer of hydride in terms of its possible application in redox biology. Moreover, we extended van Eldik’s work by adopting these species as model systems to monitor heterogeneous multiphase ligand substitution reactions at the solid-liquid interface.

## Substitution behavior

Tuning of the reactivity of Ru(II) polypyridyl complexes in terms of monodentate ligand exchange reactions that could be of biological relevance, was studied (Chrzanowska et al., 2017; Chrzanowska et al., 2018; Chrzanowska et al., 2020b; Hubbard et al., 2020). Intracellular hydrolysis has long been considered an important process in activating Ru(II) complexes and acting as a drug. It has been proposed that a common feature of many synthesized and studied Ru(II) complexes is their activation through the aquation of a monodentate ligand (Romero-Canelón and Sadler, 2013; Mital and Ziora, 2018). This process allows the metal center to coordinate with intracellular biomolecules such as DNA, proteins, and other biological targets. Metal drugs are often administered in solution, so their stability and further transformation in the human body are essential. The aquation of an intravenously administered Ru(II) complex must not be too rapid under physiological conditions, as it must be selectively activated before reaching its biological destination in cancer cells without other side reactions. In the examined compounds, ruthenium(II) is always surrounded by the tridentate N, N, N-donor ligand (terpy = 2,2′:6′,2″-terpyridine) and various bidentate N,N-donor ligands (en = ethylenediamine, ampy = 2-(aminomethyl)pyridine, tmen = N,N,N′,N′-tetramethylethylenediamine, bipy = 2,2′-bipyridine, phen = 1,10-phenantroline).

**FIGURE 1:**
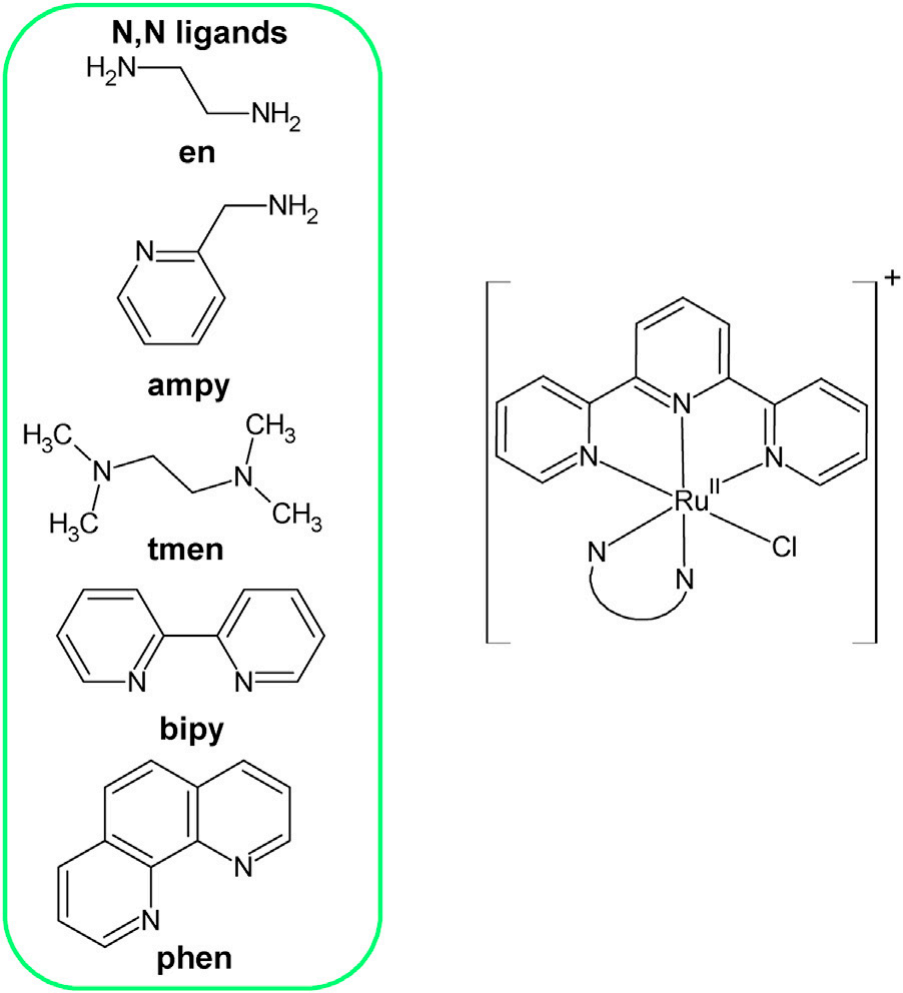
Structure of Ru(II) complexes with the general formula [Ru^II^(terpy)(N^∧^N)Cl]^+^.

These ligands are spectator ligands, very firmly bound to the central ion and do not undergo any substitution or any other chemical transformations in aqueous solutions, unlike the ligand X, which occupies the 6th coordinate position. The latter one is an actor ligand and is substituted by solvent molecules and other nucleophiles. The properties of N, N-donor spectator ligands, have a decisive influence on the rate of exchange of ligand X in a given coordination compound. Carefully thought-out selection of these ligands, having various electronic properties and providing different steric effects, allowed to obtain a series of five complexes with the general formula [Ru^II^(terpy)(N^∧^N)Cl]^+^ (where N^∧^N = N,N—donor ligands), see Figure 1. Complexes are characterized by different lability, important from the point of view of further research on their potential catalytic abilities.

The previously described studies (Chrzanowska et al., 2017; Chrzanowska et al., 2018; Chrzanowska et al., 2020b; Hubbard et al., 2020) have shown that the reactivity and acid-base properties of [Ru^II^(terpy)(N^∧^N)X]^+/2+^ complexes, where X = Cl^−^ or H_2_O, are strongly influenced by both electronic factors and steric properties of bidentate ligands. Table 1 summarizes the most important data necessary to compare the reactivity of these complexes. Aquation reactions $k_{H_{2}O}$ , anation by chloride *k*_Cl_, substitution by thiourea *k*_TU_, equilibrium constants for the chlorido complex aquation reactions *K*, and p*K*_a_ values of their aqua derivatives, determined at 25°C, are reported in Table 1.

**TABLE 1:** Values of $k_{H_{2}O}$ for [Ru (terpy) (N^∧^N)Cl]^+^ complexes, *k*_Cl_, *k*_TU_, and p*K*_a_ of [Ru (terpy) (N^∧^N) (H_2_O)]^2+^ complexes, and equilibrium constants *K* (= $k_{H_{2}O}$ /*k*_Cl_) at *T* = 25°C (Chrzanowska et al., 2017; Chrzanowska et al., 2018; Chrzanowska et al., 2020b; Hubbard et al., 2020).

N^N	en	ampy	tmen	bipy	phen
	${\left[Ru\left(terpy\right)\left(N^{\wedge }N\right)Cl\right]}^{+}+H_{2}O={\left[Ru\left(terpy\right)\left(N^{\wedge }N\right)\;\left(H_{2}O\right)\right]}^{2+}+{Cl}^{-}$				
$10^{3}\;k_{H_{2}O},\left[s^{-1}\right]$	7.0 ± 0.1	0.64 ± 0.03	0.12 ± 0.01	0.11 ± 0.01	0.072 ± 0.001
	${\left[Ru\left(terpy\right)\left(N^{\wedge }N\right)\;\left(H_{2}O\right)\right]}^{2+}+Cl^{-}={\left[Ru\left(terpy\right)\left(N^{\wedge }N\right)Cl\right]}^{+}+H_{2}O$				
$10^{3}\;k_{Cl},\left[M^{-1}s^{-1}\right]$	12.8 ± 0.4	2.09 ± 0.08	0.43 ± 0.02^a^	0.42 ± 0.01^a^	0.36 ± 0.01^a^
	${\left[Ru\left(terpy\right)\left(N^{\wedge }N\right)\;\left(H_{2}O\right)\right]}^{2+}+TU\;\to \;{\left[Ru\left(terpy\right)\left(N^{\wedge }N\right)TU\right]}^{2+}+H_{2}O$				
$10^{3}\;k_{TU},\left[M^{-1}s^{-1}\right]$	37.4 ± 0.4	3.57 ± 0.05	0.87 ± 0.03^a^	0.59 ± 0.02^a^	0.60 ± 0.02^a^
	${\left[Ru\left(terpy\right)\left(N^{\wedge }N\right)Cl\right]}^{+}+H_{2}O\;⇌\;{\left[Ru\left(terpy\right)\left(N^{\wedge }N\right)\left(H_{2}O\right)\right]}^{2+}+{Cl}^{-}$				
***K*,** [**M]**	0.62 ± 0.03	0.31 ± 0.03	0.28 ± 0.01	0.26 ± 0.03	0.20 ± 0.01
	${\left[Ru\left(terpy\right)\left(N^{\wedge }N\right)\left(H_{2}O\right)\right]}^{2+}⇌\;{\left[Ru\left(terpy\right)\left(N^{\wedge }N\right)\left(OH\right)\right]}^{+}+H^{+}$				
**p*K***_***a***_ **(*I* ≈ 0 M)**	10.83 ± 0.03	10.36 ± 0.03	10.03 ± 0.02	9.83 ± 0.03	9.59 ± 0.03

^a^ Calculated based on thermal activation parameters.

**FIGURE 2:**
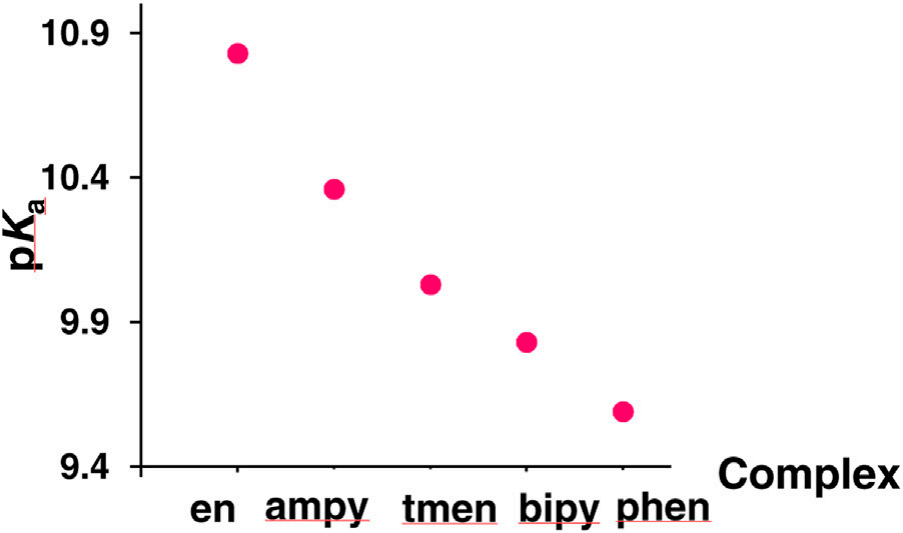
Acidic properties of examined complexes.

A careful analysis of the data contained in Table 1 indicates that the observed differences in the acidic properties of the examined complexes are consistent with the differences in their lability. The stability of the tested chlorido complexes and reactivity of its aqua derivatives change with the number of pyridine rings coordinated to the central ion, i.e., with an increase in the π-acceptor properties of the ligands. As a consequence, the rates of spontaneous aquation reactions of the [Ru^II^(terpy)(N^∧^N)Cl]^+^ complexes, as well as substitution of a coordinated water molecule in [Ru^II^(terpy)(N^∧^N)(H_2_O)]^2+^ by chloride, thiourea and N,N′-dimethylthiourea, (Spokoyny et al., 2009), increase with decreasing π-acceptor properties of N^∧^N ligands: [Ru^II^(terpy)(phen)X]^+/2+^ < [Ru^II^(terpy)(bipy)X]^+/2+^ < [Ru^II^(terpy)(ampy)X]^+/2+^ < [Ru^II^(terpy)(en)X]^+/2+^ (X = Cl^−^ or H_2_O). A π-acceptor N,N-donor ligand pulls back electron density from the Ru(II) center. A resulting increase in the electrophilicity of the metal center leads to a decrease in its reactivity. Consequently, the metal-coordinated water molecule oxygen bond is stronger in complexes with strong π-acceptor ligands, like bipyridine or phenanthroline. For that reason, the oxygen-hydrogen bond is weaker, which is manifested by the higher acidity of the aqua complexes (Figure 2).

Substitution of all hydrogen atoms of the amino groups of ethylenediamine in the most reactive complex [Ru^II^(terpy)(en)X]^+/2+^ with much larger methyl groups results in a significant decrease in the exchange rate of ligand X and the lability of [Ru^II^(terpy)(tmen)X]^+/2+^ similar to the lability of the second, most inert complex in the tested series [Ru^II^(terpy)(bipy)X]^+/2+^. A detailed analysis of the differences in the reactivity of the discussed complexes was described in our earlier review (Hubbard et al., 2020).

## Catalytic reduction of NAD^+^ to NADH

The possibility of using polypyridyl Ru(II) complexes to catalyze the reduction of NAD^+^ coenzyme to NADH in the presence of formate as a source of a hydride ion was studied (Chrzanowska et al., 2020a; Chrzanowska et al., 2022). A series of UV-Vis spectrophotometric experiments were carried out, which, among others, allowed to check the catalytic abilities of two selected, most labile and promising complexes [Ru^II^(terpy)(en)X]^+/2+^ and [Ru^II^(terpy)(ampy)X]^+/2+^. The influence of the composition of the coordination sphere, i.e., other bidentate ligands, on the mechanism of the catalytic reduction process was also examined. The first experiments of NAD^+^ reduction in the presence of Ru(II) complexes and formate ions showed that this process does not occur in an aqueous solution and requires the addition of a non-aqueous solvent. Ethyl alcohol was selected as a solvent used in medicinal procedures from several solvents that are well miscible with water. Finally, the measurements were carried out in a solution containing water and ethanol in a volume ratio of 1:9. The results of more detailed studies on the effect of the solvent on the course of the described process have been presented in earlier manuscripts (Chrzanowska et al., 2020a; Chrzanowska et al., 2022). It should be emphasized that the preliminary tests confirmed that the ethanol present in the reaction mixture is not a hydride donor, and the reduction of NAD^+^ is not a spontaneous process and does not occur without a catalyst.

**FIGURE 3:**
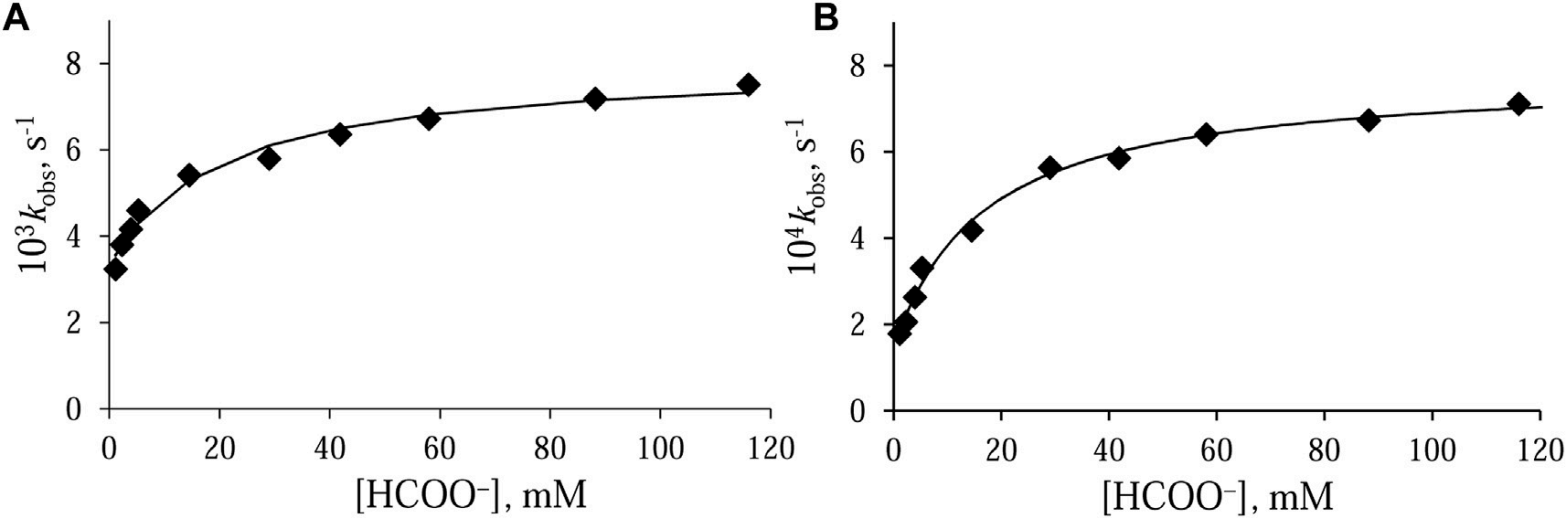
Dependence of *k*_obs_ on formate concentration for the anation of **(A)** [Ru^II^(terpy)(en)(H_2_O/EtOH)]^2+^ and **(B)** [Ru^II^(terpy)(ampy)(H_2_O/EtOH)]^2+^ in water/ethanol mixture (1:9, v/v). Experimental conditions: [Ru(II)] = 0.106 mM, *T* = 36.8°C.

Before starting the study of the catalytic process, the behavior of both [Ru^II^(terpy)(N^∧^N)(H_2_O/EtOH)]^2+^ complexes in a water/ethanol solution (1:9, v/v) containing formate anions was examined. The substitution reaction was followed as a function of formate concentration. The values of the observed rate constants, *k*_obs_, increase with increasing concentration of incoming ligand. At a high concentration of formate, the systems reach equilibrium, which means that a further increase in its concentration did not accelerate the reaction. The *k*_obs_—[HCOO^−^] relationships determined for both Ru(II) systems are characterized by a point of intersection with the ordinate axis different from zero (Figure 3). This means that in both cases, the reaction is reversible, and the reverse reaction is the substitution of the HCOO^−^ anion with a molecule of water or ethanol.

The observations can be explained by assuming that the discussed process consists of two reversible stages. The first of them leads to the formation of an ion-pair, and the product of the second is the formate derivative of the initial complex:

$${\left[Ru^{ΙΙ}\left(terpy\right)\left(N^{\wedge }N\right)\left(H_{2}O/EtOH\right)\right]}^{2+}+HCOO^{-}\begin{array}{c} Q_{1}\\ ⇌ \end{array}\begin{array}{c} \left\{{\left[{Ru}^{ΙΙ}\left(terpy\right)\left(N^{\wedge }N\right)\left(H_{2}O/EtOH\right)\right]}^{2{+}_{∎}}HCOO^{-}\right\}\\ k_{-1}↿⇂k_{1}\\ {\left[Ru^{ΙΙ}\left(terpy\right)\left(N^{\wedge }N\right)\left(HCOO\right)\right]}^{+}+H_{2}O/EtOH \end{array}$$ (1)

where $Q_{1}$ is the equilibrium constant of the precursor (ion-pair) formation process, and *k*_1_ and *k*_−1_ are the rate constants for formate anation and reverse solvolysis reactions, respectively.

The studies carried out show that the formation of formate complexes takes place on different time scales. In the range of concentrations of HCOO^−^ anions used in the study of hydride ion transfer to ${NAD}^{+}$ , the reaction lasts 8–17 min for the Ru-en complex and 1.5–6.5 h for the Ru-ampy complex. It follows that the complex with ampy reacts 11–18 times slower than its analog with en. This is consistent with the behavior of both complexes in the aqueous solution described above. Additionally, the value of the second order rate constant $k_{1}Q_{1}$ of the anation of the Ru-ampy complex is also an order of magnitude lower for the Ru-en complex. This explains the fact that formate anation of the ${\left[{Ru}^{II}\left(terpy\right)\left(ampy\right)\left(H_{2}O/EtOH\right)\right]}^{2+}$ complex is much slower than [Ru^II^(terpy)(en)(H_2_O/EtOH)]^2+^. An important observation resulting from this research is the fact that the overall process of reducing NAD^+^ to NADH, under the same measurement conditions, takes place on a much longer time scale than the formation of the formate complexes [Ru^II^(terpy)(N^∧^N)(HCOO)]^+^, by substitution of the solvent molecule in the starting complexes ${\left[{Ru}^{II}\left(terpy\right)\left(N^{\wedge }N\right)\left(H_{2}O/EtOH\right)\right]}^{2+}$ .

**FIGURE 4:**
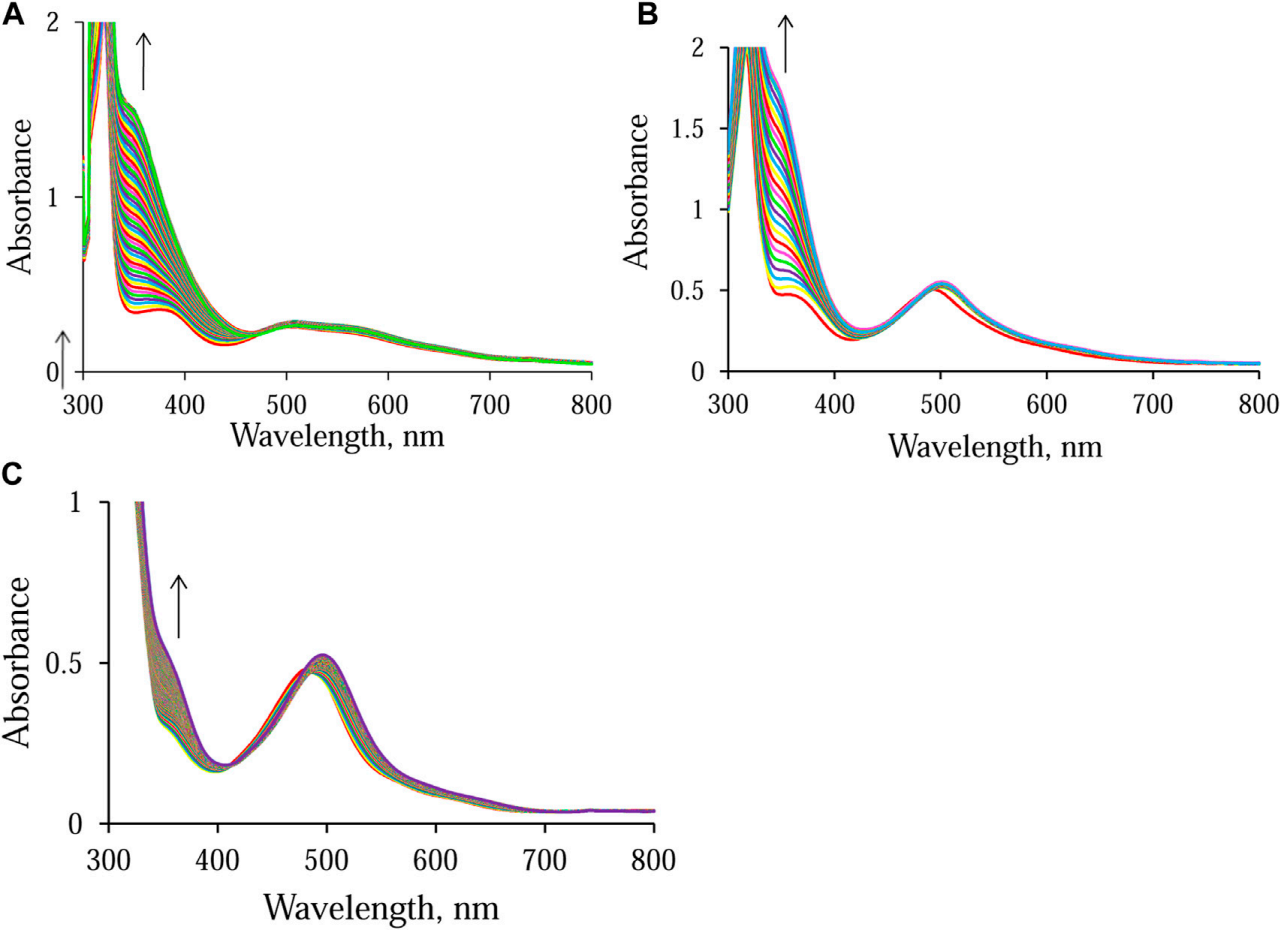
Spectral changes observed during the reduction of NAD^+^ to 1,4-NADH in the presence of formate in water/ethanol (1:9, v/v) solution catalyzed by [Ru^II^(terpy)(N^∧^N)(H_2_O/EtOH)]^2+^ monitored at 340 nm. Experimental conditions: **(A)** N^∧^N = en, [HCOO^−^] = 14.5 mM; **(B)** N^∧^N = ampy, [HCOO^−^] = 58 mM; **(C)** N^∧^N = bipy, [HCOO^−^] = 58 mM; [Ru] = 0.053 mM [NAD^+^] = 0.31 mM, *T* = 36.8°C; spectra recorded every **(A)** 15 min, **(B)** 60 min, and **(C)** 15 min.

As expected, the different electronic properties of the N, N-donor ligands, ampy and en, affect the overall conversion time of NAD^+^ to NADH. The time scale of the reaction in the presence of Ru-ampy is about twice as long as that of the reaction with Ru-en. A similar effect was observed when following the NAD^+^ reduction reaction in the presence of [Ru^II^(terpy)(bipy)(H_2_O/EtOH)]^2+^. The stronger π-back Ru(II)–2,2′-bipyridine bonding results in a decrease in the reactivity of the Ru(II) complex by many orders of magnitude. Consequently, although [Ru^II^(terpy)(bipy)(H_2_O/EtOH)]^2+^ catalyzes the reduction of NAD^+^ in the presence of formate, the process is orders of magnitude slower (Chrzanowska et al., 2022). Formation of NADH was monitored at its characteristic wavelength maximum of 340 nm. Under the selected experimental conditions, the NAD^+^ reduction reaction catalyzed by [Ru^II^(terpy)(en)(H_2_O/EtOH)]^2+^ and [Ru^II^(terpy)(ampy)(H_2_O/EtOH)]^2+^ lasts about 12 and 21 h, and is accompanied by an increase in absorbance of about 1.2 and 1.3 at 340 nm, respectively (Figures 4A, B). Whereas, during the reaction catalyzed by [Ru^II^(terpy)(bipy)(H_2_O/EtOH)]^2+^, the change in absorbance observed after about 42 h is only 0.2 (Figure 4C).

The results obtained from studies as a function of formate concentration show that formate anions not only coordinate to Ru(II) and serve as a source of a hydride ion. They also play an important role in the further stage of the entire process, catalyzing the hydride complex formation reaction [Ru^II^(terpy)(N^∧^N)(H^−^)]^+^, the actual form of the catalyst. The observations made can be explained based on the reaction mechanism presented by the equations:

$${\left[Ru^{ΙΙ}\left(terpy\right)\left(N^{\wedge }N\right)\left(HCOO\right)\right]}^{+}+HCOO^{-}\begin{array}{c} Q_{2}\\ ⇌ \end{array}\begin{array}{c} \left\{{\left[Ru^{ΙΙ}\left(terpy\right)\left(N^{\wedge }N\right)\left(HCOO\right)\right]}^{{+}_{∎}}HCOO^{-}\right\}\\ k_{2}↓\\ {\left[Ru^{II}\left(terpy\right)\left(N^{\wedge }N\right)\left(H^{-}\right)\right]}^{+}+HCOO^{-}+CO_{2} \end{array}$$ (2)

$${\left[Ru^{II}\left(terpy\right)\left(N^{\wedge }N\right)\left(H^{-}\right)\right]}^{+}+{NAD}^{+}\overset{fast}{\underset{+H_{2}O/EtOH}{\to }}{\left[Ru^{II}\left(terpy\right)\left(N^{\wedge }N\right)\left(H_{2}O/EtOH\right)\right]}^{2+}+NADH$$ (3)

The slow stage of the formation of the hydride complex [Ru^II^(terpy)(N^∧^N)(H^−^)]^+^ is preceded by a fast and reversible reaction for the production of the precursor ion-pair complex {[Ru^II^(terpy)(N^∧^N)(HCOO)]^+^▪HCOO^−^}. In the subsequent fast reaction, the hydride ion is transferred from the complex [Ru^II^(terpy)(N^∧^N)(H^−^)]^+^ to NAD^+^ coenzyme, and at the same time, the starting complex [Ru^II^(terpy)(N^∧^N)(H_2_O/EtOH)]^2+^ is reformed. Studies on the formate anation of the starting solvated complexes and the hydride anion transfer to NAD^+^, catalyzed by these complexes, have shown that under the same experimental conditions, the formation of the formato complexes is orders of magnitude faster than the formation of NADH. The total process of hydride ion transfer for both systems was also studied as a function of NAD^+^ and catalyst concentration at low and high formate concentrations. The most important conclusion from these studies is that the concentration of NAD^+^ does not affect either the pseudo-first-order rate constant describing the redox process in solutions with a low concentration of formate or the rate (pseudo-zero-order rate constant) of this process in solutions with a high concentration of formate.

Other experiments performed showed that the reduction of NAD^+^ employing tetrahydroborate (BH_4_^−^) as a hydride source is very fast compared to the process catalyzed by the ruthenium(II) complexes, in which formate is the source of the H^−^ ion. The first process takes place on a timescale of a few seconds, whereas the second takes many hours.

Additionally, studying the kinetic effects associated with isotopic substitution, which is often helpful in determining the reaction mechanism, was performed. Substitution of a given isotope with a heavier isotope of an element forming a bond that is cleaved in the activation process results in a change in the activation energy, which translates into the value of the reaction rate constant. This is called the kinetic isotope effect, KIE. Generally, the substitution of a lighter X isotope with a heavier one, e.g., substitution of protium with deuterium, results in a reduction of the rate constant of the reaction and is a strong argument supporting the postulate of breaking the bond with the participation of the X atom in the rate-limiting stage of the entire process. (Wang et al., 2014). The KIE study was used to determine the mechanism of the [Ru^II^(terpy)(N^∧^N)(H_2_O/EtOH)]^2+^ complex-catalyzed NAD^+^ reduction reaction in the presence of formate ions. For this purpose, the protium-containing sodium formate, HCOONa, was replaced with the deuterated salt, DCOONa. The use of deuterated formate for the described studies resulted in a greater decrease in the rate of the process catalyzed by [Ru^II^(terpy)(en)(H_2_O/EtOH)]^2+^ ions than [Ru^II^(terpy)(ampy)(H_2_O/EtOH)]^2+^, but values of KIE in both cases were similar to those found in the literature. This means that, according to the transition state theory, the C-H (C-D) bond cleavage takes place in the activation process. (Espenson, 1995). The slower rate *r* of the production of the NADD (reduced form of the coenzyme in the presence of isotopically labeled formate DCOO^−^ as a source of D^−^ ion) than NADH (in the presence of normal formate HCOO^−^ as a source of H^−^ ion), indicates that the formation of the hydrido complex is the rate-limiting step of the overall process.

**SCHEME 1:**
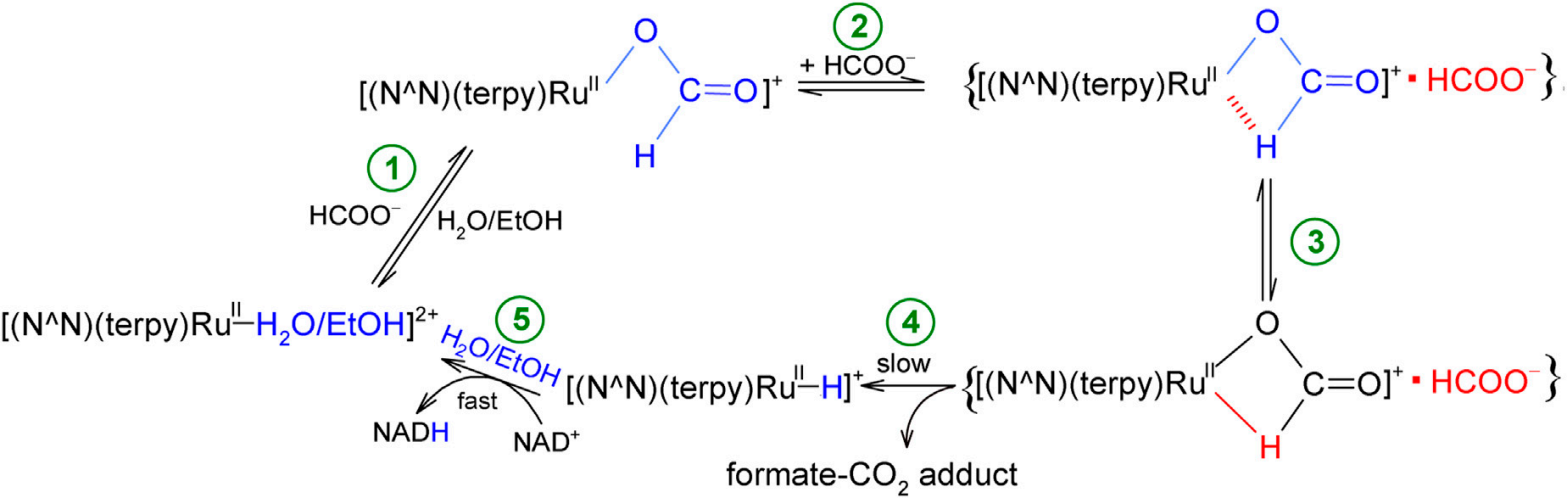
Proposed mechanism of NAD^+^ coenzyme reduction in the presence of formate, catalyzed by inorganic polypyridyl ruthenium(II) complexes.

All the described studies aimed at proposing a reaction mechanism (Scheme 1) for the reduction of NAD^+^ coenzyme to NADH, catalyzed by non-organometallic complexes of the type [Ru^II^(terpy)(N^∧^N)(H_2_O/EtOH)]^2+^.

The studied conversion of NAD^+^ to NADH coenzyme in the presence of formate begins with an anation reaction of the starting solvated complex [Ru^II^(terpy)(N^∧^N)(H_2_O/EtOH)]^2+^, the product of which is its formato derivative [Ru^II^(terpy)(N^∧^N)(HCOO)]^+^ (step 1). In the formed complex, the formate anion is coordinated with the central ion through an oxygen atom. Then, in the presence of a large concentration of formate, an ion-pair (outer-sphere complex) is generated, which consists of the formate complex and the HCOO^−^ anion, {[Ru^II^(terpy)(N^∧^N)(HCOO)]^+^▪HCOO^−^} (step 2). The formate anion occupying a place in the outer coordination sphere plays a crucial role in the whole process, as it induces the rearrangement in the inner-coordination sphere of the complex. This involves twisting (isomerization) of the formate ion and changing its coordination mode. In this way, a complex with the formate anion bound to the coordination center by oxygen and hydrogen atoms is produced (step 3). Then, the chelating HCOO^−^ undergoes decarboxylation, and the released carbon dioxide molecule leaves the system together with the formate anion in the immediate surroundings of the central ion (stage 4). As a consequence, the hydrido complex [Ru^II^(terpy)(N^∧^N)(H^−^)]^+^ is formed, which is a donor of a hydride anion, transferred to NAD^+^ coenzyme to produce NADH in the subsequent step. Concomitantly, the hydride ligand leaving the inner-coordination sphere of the Ru(II) center, is replaced by a solvent molecule H_2_O or EtOH (step 5). As a result, the catalyst is reformed in its original form, [Ru^II^(terpy)(N^∧^N)(H_2_O/EtOH)]^2+^, and the catalytic cycle can repeat itself. Although the electronic and steric properties of N,N-donor ligands affect the catalytic activity of the studied complexes, they do not change the mechanism of the catalytic cycle of NAD^+^ to NADH conversion.

## Interaction of nanoparticles and nanoscale coordination compounds at the interface of multiphase chemical and medicinal related processes

As a continuation of the research described above, we have performed experiments that will help us to find an answer to the key question, what do we know about the mechanism of ligand displacement reactions occurring on the surfaces of ruthenium(II) coordination compounds at the nano and submicron scale. As described above, once introduced into the system, coordination compounds can rapidly interact with many biomolecules. These interactions may include redox or substitution reactions that directly affect the inactivation process and, thus their bioavailability and distribution. The transformation of complexes to the nano or submicron scale can affect the stability and behavior of such compounds in aqueous solutions. Surface ligands can play an important role in tuning the functional properties of metal nanomaterials in terms of binding properties, electronic structure, and chemical transformations. Experimental work, therefore, focused on the possibility of changing the structure and redox properties of NPs and NSCCs by systematically replacing coordinated ligands with stronger nucleophiles induced at the solid-liquid interface in multiphase chemical reactions. Ligand substitutions within NSCCs can be induced *in situ* to modify their solid-state chemical properties. Therefore, emphasis was placed not only on studying the surface interactions of NSCC with potential ligands but also on going a step further to induce ligand substitution and redox reactions within the NSCC and modifying their structure and reactivity in a similar way as for homogeneous coordination chemistry. Applying this basic approach could lead to far-reaching advances in tuning the chemical reactivity and surface properties of NPs and NSCCs.

**SCHEME 2:**
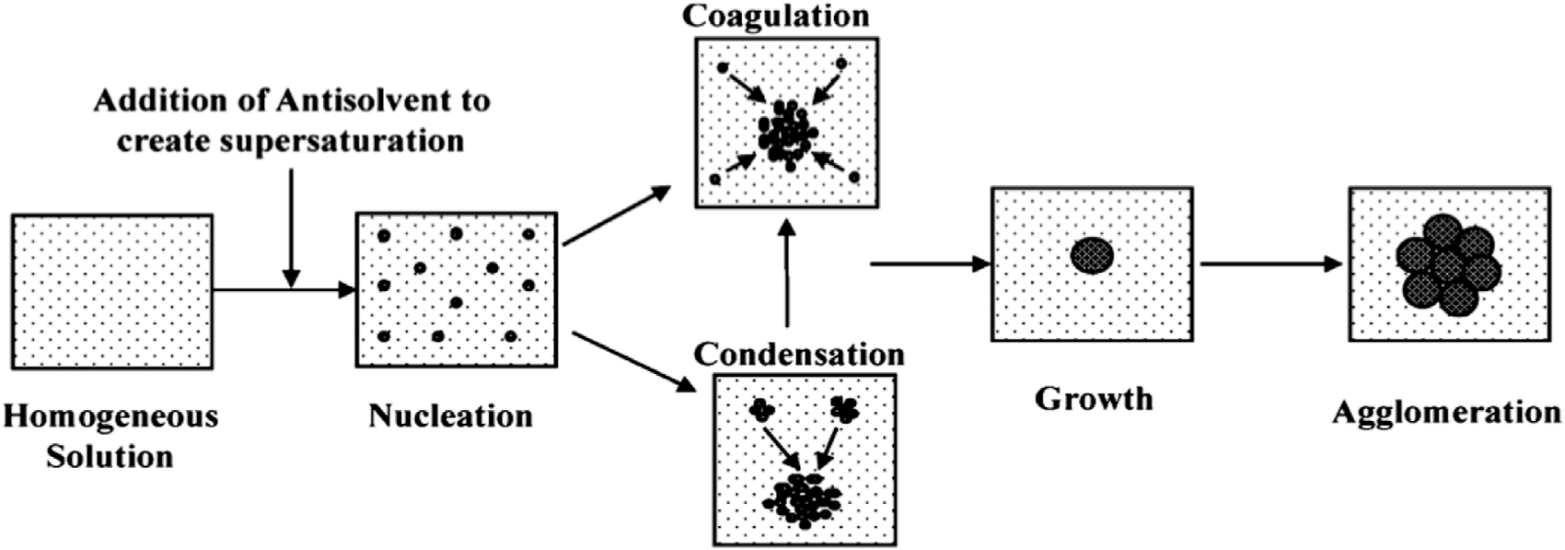
Schematic diagram of the particle precipitation process.

We synthesized colloidal coordination compounds in the submicron range from starting complexes *via* the anti-solvent procedure. Anti-solvent precipitation combined with ultrasonic and centrifugation techniques were used to synthesize NP and NSCC, as shown above (Scheme 2). The addition of a suitable surfactant prevents agglomeration and promotes the formation of smaller particles with larger submicron surface areas.

**FIGURE 5:**
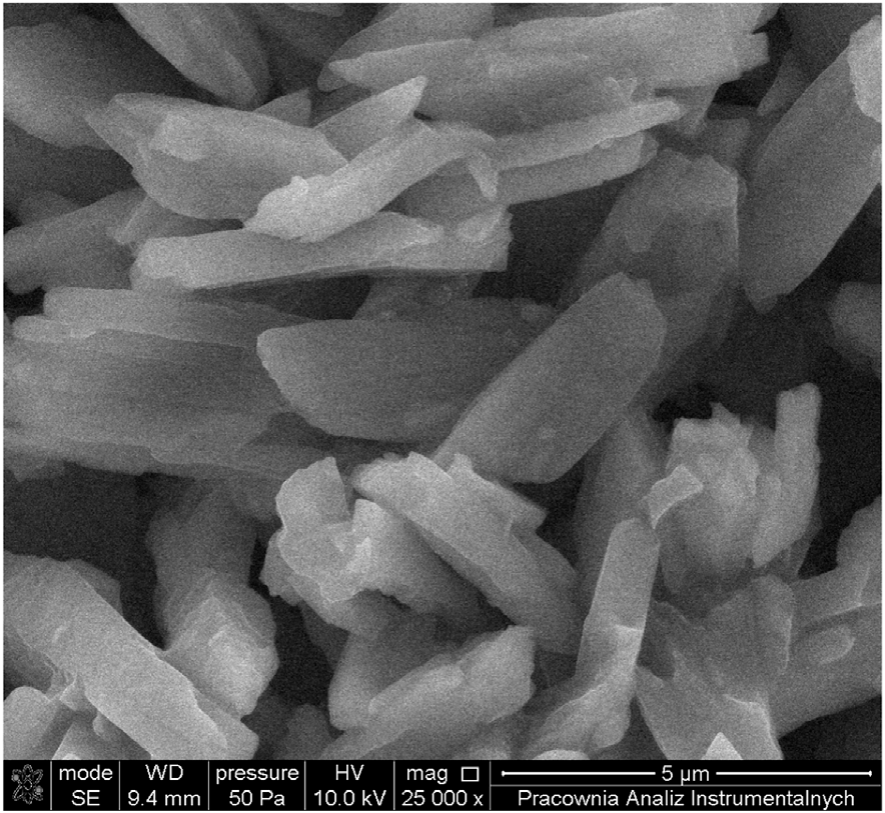
SEM image for particles synthesized from the complex [Ru(terpy)(en)(H_2_O)]^2+^ *via* the anti-solvent process stabilized with a 0.2 mM Tween 20 solution.

Particles size was estimated by DLS and SEM methods (Figure 5). Systematic studies of ligand substitution reactions on such solid-state Ru(II)-polypyridyl complexes will now be undertaken as a function of anti-solvent, sonication time, nucleophilicity and concentration of introduced ligand, type, and concentration of a surfactant, pH, temperature and reaction time. Substitution events can be monitored by UV-Vis and EDX spectroscopies.

Understanding nano-bio interactions can contribute to the advancement of nanomedicine. Substitution and redox reactions are factors that regulate the performance and toxicity of nanomaterials. A mechanistic explanation of the chemical processes at the solid-liquid interface could have far-reaching implications for systematically fine-tuning the properties of NSCCs and their interactions with nanoparticles and molecules of biological and catalytic importance. Such knowledge is also essential for understanding the advantages and disadvantages of using specific materials and can help to improve existing catalysts or design new ones (Liu et al., 2017; Langford et al., 2018).

## Data Availability

The raw data supporting the conclusion of this article will be made available by the authors, without undue reservation.
